# Describing organ dysfunction in the intensive care unit: a cohort study of 20,000 patients

**DOI:** 10.1186/s13054-019-2459-9

**Published:** 2019-05-23

**Authors:** Andrea Soo, Danny J. Zuege, Gordon H. Fick, Daniel J. Niven, Luc R. Berthiaume, Henry T. Stelfox, Christopher J. Doig

**Affiliations:** 1 Department of Critical Care Medicine, University of Calgary, McCaig Tower, Ground Floor, 3134 Hospital Drive NW, Calgary, Alberta T2N 5A1 Canada; 2 Department of Community Health Sciences, Cumming School of Medicine, University of Calgary, 3280 Hospital Drive NW, Calgary, Alberta T2N 4Z6 Canada

**Keywords:** Multiple organ failure, Cohort studies, Natural history, Intensive care units, Organ dysfunction scores, Critical illness

## Abstract

**Background:**

Multiple organ dysfunction is a common cause of morbidity and mortality in intensive care units (ICUs). Original development of the Sequential Organ Failure Assessment (SOFA) score was not to predict outcome, but to describe temporal changes in organ dysfunction in critically ill patients. Organ dysfunction scoring may be a reasonable surrogate outcome in clinical trials but further exploration of the impact of case mix on the temporal sequence of organ dysfunction is required. Our aim was to compare temporal changes in SOFA scores between hospital survivors and non-survivors.

**Methods:**

We performed a population-based observational retrospective cohort study of critically ill patients admitted from January 1, 2004, to December 31, 2013, to 4 multisystem adult intensive care units (ICUs) in Calgary, Canada. The primary outcome was temporal changes in daily SOFA scores during the first 14 days of ICU admission. SOFA scores were modeled between hospital survivors and non-survivors using generalized estimating equations (GEE) and were also stratified by admission SOFA (≤ 11 versus > 11).

**Results:**

The cohort consisted of 20,007 patients with at least one SOFA score and was mostly male (58.2%) with a median age of 59 (interquartile range [IQR] 44–72). Median ICU length of stay was 3.5 (IQR 1.7–7.5) days. ICU and hospital mortality were 18.5% and 25.5%, respectively. Temporal change in SOFA scores varied by survival and admission SOFA score in a complicated relationship. Area under the receiver operating characteristic (ROC) curve using admission SOFA as a predictor of hospital mortality was 0.77. The hospital mortality rate was 5.6% for patients with an admission SOFA of 0–2 and 94.4% with an admission SOFA of 20–24. There was an approximately linear increase in hospital mortality for SOFA scores of 3–19 (range 8.7–84.7%).

**Conclusions:**

Examining the clinical course of organ dysfunction in a large non-selective cohort of patients provides insight into the utility of SOFA. We have demonstrated that hospital outcome is associated with both admission SOFA and the temporal rate of change in SOFA after admission. It is necessary to further explore the impact of additional clinical factors on the clinical course of SOFA with large datasets.

**Electronic supplementary material:**

The online version of this article (10.1186/s13054-019-2459-9) contains supplementary material, which is available to authorized users.

## Introduction

Multiple organ dysfunction syndrome (MODS)—also variably described as multiple organ failure or multisystem organ failure—is common in intensive care unit (ICU) patients [1]. It is often present at the time of ICU admission and/or present at time of ICU death. MODS has been described as a process of sequential and progressive organ dysfunction and/or failure that precedes death, its presence not infrequently influencing discussions and decisions to limit or withdraw life-sustaining interventions [2–4]. The pathophysiology of MODS remains poorly elucidated [1, 5–8]. In the absence of clearly defined pathophysiology for MODS, clinical diagnostic scoring systems have been developed to assist diagnosis, to describe progression, and to assess prognosis [9].

Though multiple scoring systems have been developed, the most common in practical use today is the Sequential Organ Failure Assessment (SOFA) score [10–12]. It was developed by consensus, in contrast to other scores that used statistical techniques to develop models (Logistic Organ Dysfunction System, Multiple Organ Dysfunction Score) [13]. The SOFA score, as with other organ dysfunction scoring systems, does not differentiate between chronic organ dysfunction present due to underlying disease, and the effects of acute organ dysfunction related to critical illness [14].

The original article reporting the development of the SOFA score described two main roles for the score: (1) to describe the natural history of organ dysfunction and (2) to assess the effects of new therapies. SOFA scores have been reported in many clinical trials in critical care [15–17]. Many studies have examined the use of SOFA to predict mortality in critically ill patients [17–21]. As such, some investigators have suggested that SOFA scores can be used to inform triage decisions during disaster management [22–26]. Some research has suggested modifications to the SOFA score for specific ICU patient populations such as trauma patients, patients with traumatic brain injury, neurocritical care patients, and many other conditions [27–30]. Few articles have described the clinical course of organ dysfunction using SOFA scores in populations of ICU patients, one of the original purported uses of such a score [18, 20, 31–34].

The objective of our study was to describe the clinical course of organ dysfunction among a heterogeneous cohort of critically ill patients admitted to multisystem adult ICUs.

## Methods

The study was approved by the University of Calgary Conjoint Health Research Ethics Board, and a waiver of consent was granted (REB15-2010). This was a population-based observational retrospective cohort study, consisting of all consecutive critically ill adult patients (≥ 18 years) admitted to one of the four multisystem ICUs in Calgary, Canada, from January 1, 2004, to December 31, 2013. Patients were excluded if they were admitted to a coronary care unit, had undergone cardiac surgery, or survived ICU with a length of stay < 48 h after elective surgery. In the acute care hospitals in Calgary, some have surgical step-down or high observation units that admit elective postoperative patients for ‘routine’ monitoring. Therefore, patients admitted to ICU for postoperative monitoring following uncomplicated elective surgery with an ICU length of stay in the ICU < 48 h were excluded [19].

.

Demographic, clinical, severity of illness, diagnostic, and outcomes data were collected prospectively using an electronic medical record (TRACER). The details of this database have been described elsewhere [35, 36]. In summary, TRACER is a longitudinal database incorporating clinical and outcome data derived from electronic health records for adult ICU patients in the Calgary area. Data is received from various source systems, including the Quantitative Sentinel (GE-Marquette Medical) and eCritical MetaVision (*i*MD*soft*) clinical information systems, Millennium (Cerner Millenium Pathnet) and Sunquest (Sunquest Information) laboratory information systems, and Alberta Health Services corporate databases. This data repository exists to provide data for research, quality and process improvement, as well as to enhance the provision of care to the critically ill.

Only the patient’s first admission to the ICU during the study period and the first 14 days of a patient’s ICU stay was considered. Patients were followed from the day of first ICU admission until discharge from ICU, end of the first 14 days of ICU stay, or death, which ever occurred first.

The calculation of the SOFA score requires the determination every 24 h of the most abnormal physiologic, laboratory, or clinical data within each of the six organ systems (central nervous system (CNS), respiratory, hepatic, renal, coagulation, and cardiovascular). Daily calculation of CNS dysfunction was based on the Glasgow Coma Scale (GCS) which was determined daily by the attending physician as either the actual GCS, in the absence of sedating medications, or the best estimate of GCS without the effects of sedation medications if they were being used. A score from 0 (normal) to 4 (most abnormal) was assigned for each organ system for a minimum SOFA score of 0 to a maximum SOFA score of 24. We followed the exact definitions of the original publication and have previously validated our collection including an audit and manual validation [10, 31]. For each patient, the admission SOFA score was calculated based on the first 24 h of data (therefore if a patient was admitted at 02:00 am, the admission SOFA would be based on the 02:00 am to 01:59 am time period), and thereafter, scores were calculated for each subsequent patient day based on the 07:00 am to 06:59 am time frame. With this method, there may be overlap in data used to calculate the admission SOFA score and the second SOFA score. If a patient was admitted between midnight and 6:59 am, a daily SOFA score in addition to the admission SOFA was not calculated for the short time period between the admission time and 6:59 am. Among all analyzed patient days, 14.5% of patient days were missing a daily SOFA score; however, the majority (81.8%) of those days were the last day of the patient’s stay (with patients most often being in ICU less than half a day on their last day) and would therefore be unlikely to alter results significantly. Missing values were not imputed. For each patient, we also calculated the maximum SOFA score which was the sum of the highest individual organ scores irrespective of day collected and the highest SOFA score which was the highest cumulative 24 h score collected during a patient’s ICU stay.

### Statistical analysis

Baseline characteristics were summarized with medians with interquartile ranges (IQR) for continuous data and frequencies with proportions for categorical data. For the primary objective, marginal models for longitudinal data were developed using generalized estimating equations (GEE) to model the population-averaged change in mean daily SOFA score within the first 14 days of ICU stay between survivors and non-survivors. Subgroup analyses were performed using strata defined by admission SOFA (≤ 11 vs. > 11). We stratified admission SOFA at a value of 11 because prior studies have suggested a high mortality for those with an admission SOFA > 11, including Ferreira et al. who showed a predicted mortality for admission SOFA > 11 of 95% [18] and Anami et al. who observed a mortality of 89% for admission SOFA > 11 [37]. We conducted additional subgroup analyses examining temporal changes in practice over time using GEE models comparing patients admitted in 2004–2008 to patients admitted in 2009–2013 and by ICU LOS (< 3 days versus 3 to < 7 days versus ≥7 days). Due to mean daily SOFA scores potentially following a nonlinear trend, a piecewise linear GEE model with a threshold of 8 was fitted to allow different rates of change in SOFA for days 1–7 and days 8–14. A threshold of 8 was used based on observing similar results from (1) a nonlinear GEE model; (2) a GEE model using splines with knots at days 2, 3, 5, 7, 9, 11, and 13; and (3) a GEE model with day entered a categorical variable. An exchangeable within-group correlation structure and a robust estimator of variance were assumed. Hospital mortality percentages by early change in daily SOFA scores were compared using chi-squared tests. Area under the receiver operating characteristic (ROC) curve and precision-recall (PR) curve as well as the Brier score were used to quantify the predictive ability of SOFA scores for hospital mortality. Area under the ROC curves were compared using the method described in DeLong et al. [38]. Selected sensitivity analyses were repeated for ICU mortality. Analyses were conducted in R (version 3.5.1), a language and environment for statistical computing and graphics (The R Project for Statistical Computing, https://www.r-project.org) [39]. We used R packages geepack (function: geeglm) [40], doBy (function: esticon) [41], ROCR (functions: performance and prediction) [42], pROC (functions: roc and roc.test) [43], and PRROC (functions: roc.curve and pr.curve) [44, 45].

## Results

There were 20,068 adult patients admitted to ICUs in Calgary, Canada, between January 1, 2004, and December 31, 2013, among which 61 (0.3%) patients did not have at least one SOFA score recorded and were excluded from the analysis cohort. All 61 of these patients had an ICU length of stay < 7 h with 55 (90.2%) dying in hospital, of which 53 (96.4%) died in ICU. Demographics of the remaining cohort are shown in Table 1. Of the 20,007 patients with at least 1 measured SOFA score, 42% were female and median age was 59 (IQR 44–72) years. Median ICU and hospital length of stay were 3.5 (IQR 1.7–7.5) days and 14 (IQR 6–30) days, respectively. ICU mortality was 18.4% and hospital mortality was 25.2%.

**Table 1:** Patient demographics

Characteristic^1^	All patients	Hospital survivors	Hospital non-survivors
Number of patients	20,007	14,915	5092
Sex, male	11,647 (58.2)	8738 (58.6)	2909 (57.1)
Age	59 (44–72)	56 (41–69)	67 (54–77)
Diagnostic category			
Respiratory	5443 (27.2)	4242 (28.4)	1201 (23.6)
Cardiovascular	4479 (22.4)	2858 (19.2)	1621 (31.8)
Neurologic	3049 (15.2)	2130 (14.3)	919 (18.0)
Gastrointestinal	2349 (11.7)	1700 (11.4)	649 (12.7)
Trauma	1973 (9.9)	1660 (11.1)	313 (6.1)
Metabolic/renal/poisoning	1073 (5.4)	1001 (6.7)	72 (1.4)
Other/unknown	1641 (8.2)	1324 (8.9)	317 (6.2)
Admission type			
Elective post-surgery	1139 (5.7)	949 (6.4)	190 (3.7)
Emergent post-surgery	4457 (22.3)	3488 (23.4)	969 (19.0)
No surgery	14,279 (71.4)	10,399 (69.7)	3880 (76.2)
Unknown	132 (0.7)	79 (0.5)	53 (1.0)
APACHE II score on ICU admission	18 (13–25)	16 (12–21)	26 (21–33)
GCS on ICU admission	15 (11–15)	15 (13–15)	13 (5–15)
Admission SOFA score	6 (4–10)	6 (3–8)	10 (7–13)
Respiratory SOFA	2 (0–3)	2 (0–3)	3 (2–4)
Liver SOFA	0 (0–0)	0 (0–0)	0 (0–1)
Renal SOFA	0 (0–2)	0 (0–1)	1 (0–3)
Coagulation SOFA	0 (0–1)	0 (0–1)	0 (0–2)
Cardiovascular SOFA	1 (1–4)	1 (1–3)	4 (1–4)
CNS SOFA	1 (0–2)	0 (0–2)	2 (0–4)
Maximum SOFA score^2^	8 (5–12)	7 (4–10)	13 (9–16)
Highest SOFA score^3^	7 (5–10)	6 (4–9)	11 (8–15)
ICU length of stay (days)	3.5 (1.7–7.5)	3.6 (1.9–7.4)	3.0 (1.0–7.8)
ICU mortality	3710 (18.5)	N/A	3710 (72.9)
Hospital length of stay (days)	13.9 (6.1–29.9)	16.0 (8.1–33.7)	7.1 (2.1–19.1)
Hospital mortality	5092 (25.5)	N/A	5092 (100.0)

*APACHE* acute physiology and chronic health evaluation, *SOFA* Sequential Organ Failure Assessment, *GCS* Glasgow Coma Scale, *CNS* central nervous system, *N/A* not applicable

^1^Categorical data presented as frequency (%) and continuous data presented as median with interquartile range

^2^Calculated as the sum of the highest individual organ scores irrespective of day collected

^3^Calculated as the highest cumulative 24 h score collected during the patient’s ICU stay

The median admission SOFA score among hospital survivors and non-survivors were 6 (IQR 3–8) and 10 (IQR 7–13), respectively (Table 1). The most common admission diagnostic category among survivors was respiratory (28.4%) while it was cardiovascular for non-survivors (31.8%). The greatest difference in single organ dysfunction at admission between survivors and non-survivors was in the CNS and cardiovascular systems, followed by respiratory and renal systems. The median maximum SOFA score and highest SOFA were 7 (IQR 4–10) and 6 (IQR 4–9) for survivors and 13 (IQR 9–16) and 11 (IQR 8–15) for non-survivors. The highest SOFA most often occurred on day 1 or 2 for survivors and days 1–3 for non-survivors.

**Fig. 1:**
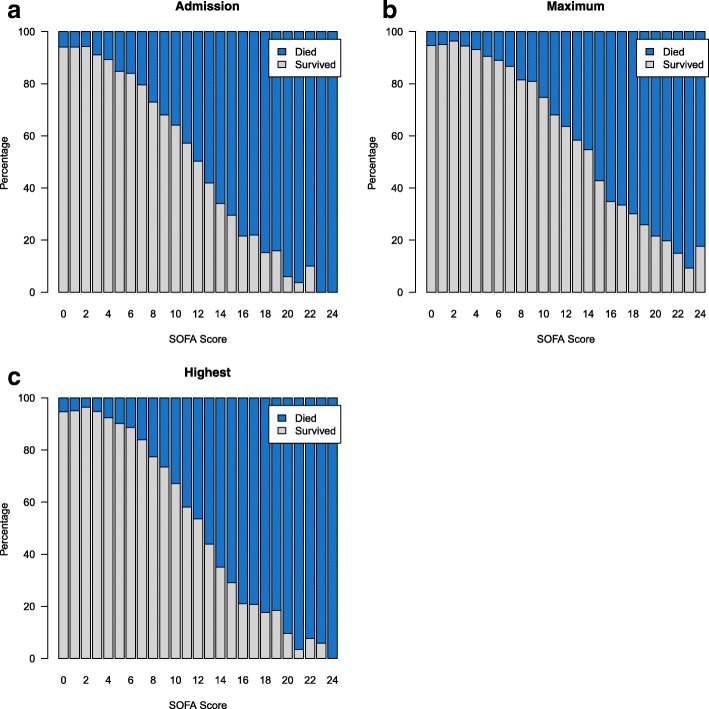
Hospital mortality by **a** admission, **b** maximum, and **c** highest SOFA scores. SOFA, Sequential Organ Failure Assessment

**Fig. 2:**
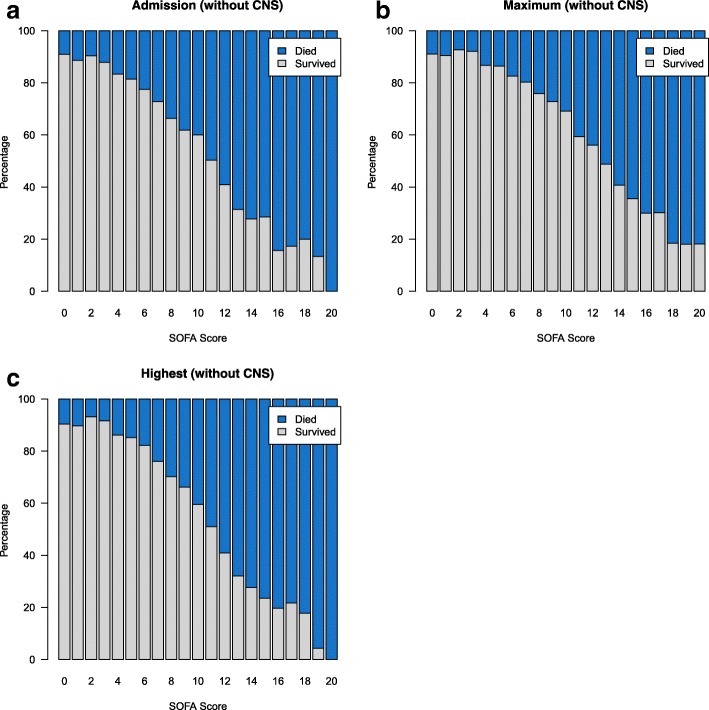
Hospital mortality by **a** admission, **b** maximum, and **c** highest SOFA scores excluding CNS component. CNS, central nervous system; SOFA, Sequential Organ Failure Assessment

Figure 1 demonstrates mortality for admission, highest, and maximum SOFA scores. Across all 3 measures of organ failure, an approximately linear increase was observed with the exception of the extremes of low and high scores. The hospital mortality rate was 5.6% for patients with an admission SOFA score of 0–2. Not surprisingly, very high scores (20–24) at admission were associated with very low survival (5.6%), but not certainty of death. There was an approximately linear increase in hospital mortality for SOFA scores of 3–19 (range 8.7% to 84.7%). Patients with significant organ dysfunction with a highest SOFA score of 15–19 had a survival of 22.9%. When the CNS component was excluded, a similar trend was observed suggesting overestimation of organ failure due to GCS as suggested in previous work [46] was unlikely (Fig. 2).

**Fig. 3:**
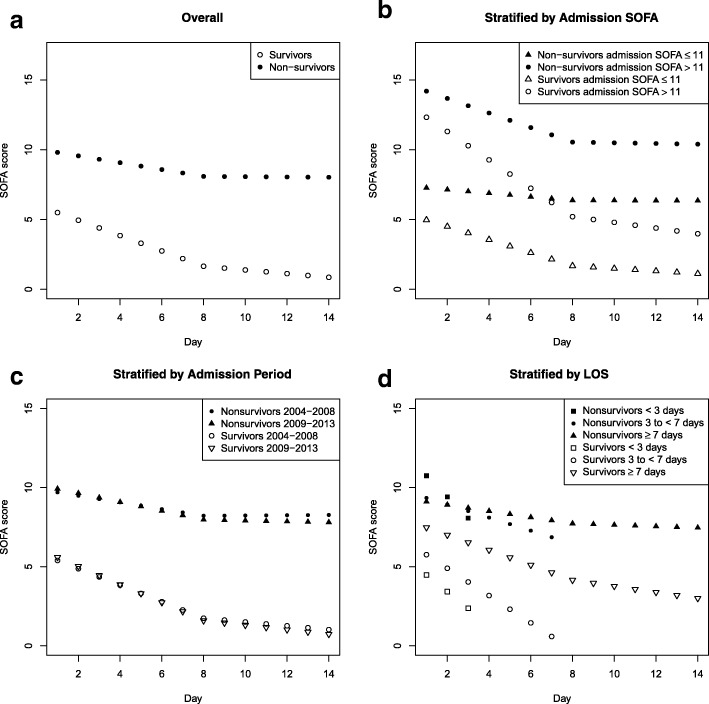
**a**–**d** SOFA scores by hospital mortality overall and by subgroups (admission SOFA, period, and LOS). SOFA, Sequential Organ Failure Assessment; LOS, length of stay

**Fig. 4:**
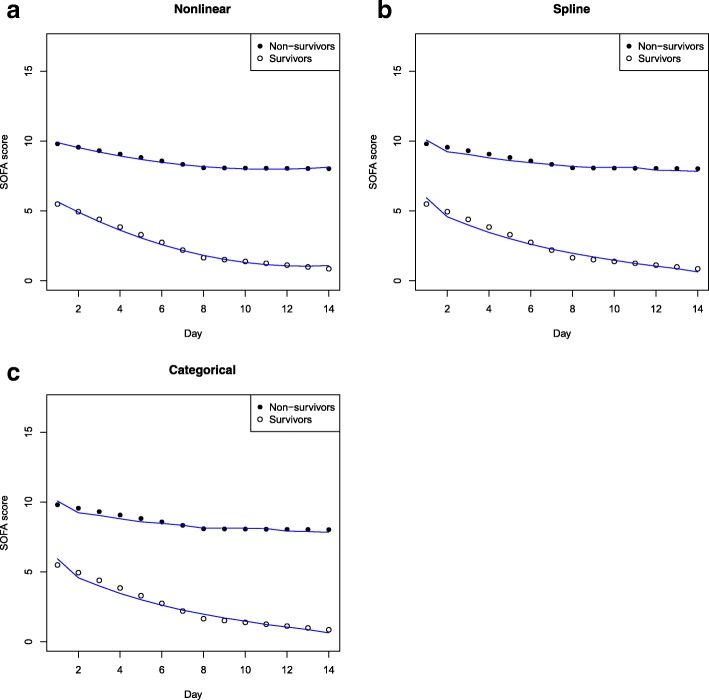
**a**–**c** SOFA scores by hospital mortality overall using **a** nonlinear, **b** spline, and **c** categorical GEE models. The points represent results from the piecewise linear GEE model. SOFA, Sequential Organ Failure Assessment

**Fig. 5:**
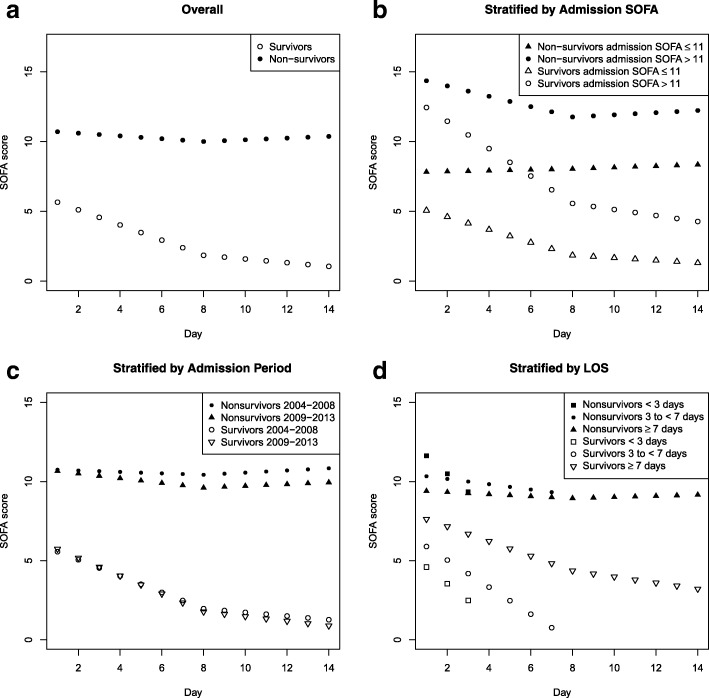
**a**–**d** SOFA scores by ICU mortality overall and by subgroups (admission SOFA, period, and LOS). SOFA, Sequential Organ Failure Assessment; LOS, length of stay

Figure 3 illustrates the clinical course of SOFA for survivors and non-survivors overall and stratified by admission SOFA, admission period, and ICU LOS. Table 2 shows that among those with admission SOFA > 11 during days 1–7 of ICU stay, rate of change in SOFA per day was − 1.02 (95% CI − 1.06, − 0.98) for survivors (*p* < 0.001) and − 0.52 (95% CI − 0.57, − 0.47) for non-survivors (*p* < 0.001) while for those with admission SOFA ≤ 11, it was − 0.47 (95% CI − 0.48, − 0.46) for survivors (*p* < 0.001) and − 0.13 (95% CI − 0.16, − 0.10) for non-survivors (*p* < 0.001). In non-survivors, we were unable to detect a change in SOFA score beyond day 7, whereas survivors had a slower but continued progressive improvement in organ function. The clinical course of SOFA for patients admitted in 2004–2008 and those admitted in 2009–2013 showed similar results (Fig. 3c, Table 2). Among those with an ICU LOS ≥ 7 days, similar trends were shown when compared to the overall cohort (Fig. 3d, Table 2). Nonlinear quadratic, spline, and categorical GEE models showed similar trends to the piecewise linear GEE models (Fig. 4). Figure 5 illustrates the clinical course of SOFA for ICU survivors compared to ICU non-survivors.

**Table 2:** Change in daily SOFA score for hospital survivors and non-survivors overall and by subgroups

		Mean rate of change in SOFA score per day (95% CI)			
		Hospital survivors		Hospital non-survivors	
		Days 1–7	Days 8–14	Days 1–7	Days 8–14
	Overall	− 0.55 (− 0.56, − 0.54) *p* < 0.001	− 0.13 (− 0.15, − 0.11) *p* < 0.001	− 0.25 (− 0.27, − 0.22) *p* < 0.001	− 0.01 (− 0.05, 0.03) *p* = 0.64
Admission SOFA score	≤ 11	− 0.47 (− 0.48, − 0.46) *p* < 0.001	− 0.09 (− 0.12, − 0.07) *p* < 0.001	− 0.13 (− 0.16, − 0.10) *p* < 0.001	− 0.004 (− 0.05, 0.04) *p* = 0.87
	> 11	−1.02 (− 1.06, − 0.98) *p* < 0.001	− 0.20 (− 0.26, − 0.15) *p* < 0.001	− 0.52 (− 0.57, − 0.47) *p* < 0.001	− 0.02 (− 0.11, 0.06) *p* = 0.58
Admission period	2004–2008	− 0.52 (− 0.54, − 0.50) *p* < 0.001	− 0.12 (− 0.15, − 0.09) *p* < 0.001	− 0.21 (− 0.25, − 0.17) *p* < 0.001	0.01 (− 0.05, 0.07) *p* = 0.78
	2009–2013	− 0.57 (− 0.59, − 0.55) *p* < 0.001	− 0.14 (− 0.17, − 0.11) *p* < 0.001	− 0.28 (− 0.32, − 0.24) *p* < 0.001	− 0.03 (− 0.09, 0.03) *p* = 0.37
ICU length of stay (days)	< 3	− 1.05 (− 1.11, − 1.00) *p* < 0.001	N/A	− 1.34 (− 1.53, − 1.15) *p* < 0.001	N/A
	3 to < 7	− 0.86 (− 0.89, − 0.84) *p* < 0.001	N/A	− 0.41 (− 0.48, − 0.35) *p* < 0.001	N/A
	≥ 7	− 0.48 (− 0.49, − 0.46) *p* < 0.001	−0.19 (− 0.21, − 0.17) *p* < 0.001	− 0.20 (− 0.23, − 0.17) *p* < 0.001	− 0.04 (− 0.09, 0.00) *p* = 0.04

*SOFA* Sequential Organ Failure Assessment, *CI* confidence interval, *N/A* Not applicable

**Fig. 6:**
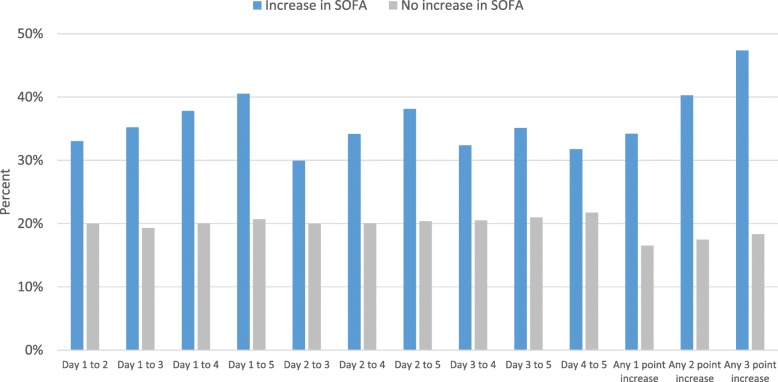
Hospital mortality by early change in daily SOFA scores. SOFA, Sequential Organ Failure Assessment

**Fig. 7:**
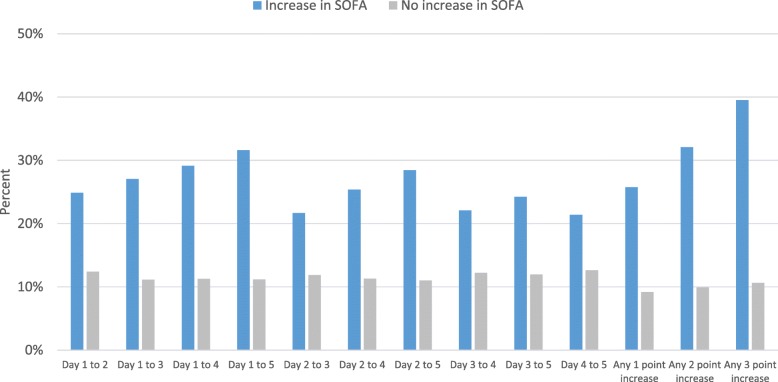
ICU mortality by early change in daily SOFA scores. SOFA, Sequential Organ Failure Assessment

Among patients with at least 2 SOFA scores, 3502 (22.2%) patients had an increase in their SOFA score from admission of ≥ 2 points occurring at any time during their ICU admission. Hospital mortality of patients who experienced this ≥ 2 point increase was significantly higher when compared to those who did not (40.3% vs 17.5%, Χ^2^ = 811, *p* < 0.001) (Table 3, Fig. 6, Additional file 1: Table S1). Similarly, ICU mortality of patients who experienced this increase was significantly higher than those who did not (32.1% vs 9.9%, *χ*^2^ = 1061, *p* < 0.001) (Table 4, Fig. 7, Additional file 1: Table S1). Additionally, patients with an increase from days 1 to 5 had a significantly higher hospital and ICU mortality than those who did not (hospital mortality: 40.5% vs. 20.7%, *χ*^2^ = 257, *p* < 0.001; ICU mortality: 31.6% vs. 11.2%, *χ*^2^ = 398, *p* < 0.001).

**Table 3:** Hospital mortality by early change in daily SOFA scores

Change in SOFA scores^1^	Number of patients	Hospital mortality, *n* (%)		
		Increase in SOFA score	No increase in SOFA score	*p* value^4^
Day 1 to day 2	15,736	1014 (33.0)	2532 (20.0)	< 0.001
Day 1 to day 3	12,650	870 (35.2)	1964 (19.3)	< 0.001
Day 1 to day 4	10,206	698 (37.8)	1678 (20.1)	< 0.001
Day 1 to day 5	8326	586 (40.5)	1424 (20.7)	< 0.001
Day 2 to day 3	12,648	913 (29.9)	1920 (20.0)	< 0.001
Day 2 to day 4	10,203	794 (34.2)	1581 (20.1)	< 0.001
Day 2 to day 5	8324	670 (38.1)	1339 (20.4)	< 0.001
Day 3 to day 4	10,205	771 (32.4)	1605 (20.5)	< 0.001
Day 3 to day 5	8326	652 (35.1)	1358 (21.0)	< 0.001
Day 4 to day 5	8326	631 (31.8)	1379 (21.8)	< 0.001
Day 1 to any day during ICU stay (any increase)	15,740	1835 (34.2)	1712 (16.5)	< 0.001
Day 1 to any day during ICU stay (≥ 2 point increase)^2^	15,740	1410 (40.3)	2137 (17.5)	< 0.001
Day 1 to any day during ICU stay (≥ 3 point increase)^3^	15,740	1082 (47.4)	2465 (18.3)	< 0.001

*SOFA* Sequential Organ Failure Assessment

^1^Based on patients who had SOFA scores on both days

^2^Comparison is patients with an increase in SOFA score ≥ 2 vs. patients with an increase < 2 points or a decrease

^3^Comparison is patients with an increase in SOFA score ≥ 3 vs. patients with an increase < 3 points or a decrease

^4^*p* value is based on a chi-squared test

**Table 4:** ICU mortality by early change in daily SOFA scores

Change in SOFA scores^1^	Number of patients	ICU mortality, *n* (%)		
		Increase in SOFA score	No increase in SOFA score	*p* value^4^
Day 1 to day 2	15,736	764 (24.9)	1571 (12.4)	< 0.001
Day 1 to day 3	12,650	669 (27.1)	1134 (11.1)	< 0.001
Day 1 to day 4	10,206	538 (29.1)	943 (11.3)	< 0.001
Day 1 to day 5	8326	457 (31.6)	768 (11.2)	< 0.001
Day 2 to day 3	12,648	661 (21.7)	1141 (11.9)	< 0.001
Day 2 to day 4	10,203	590 (25.4)	890 (11.3)	< 0.001
Day 2 to day 5	8324	500 (28.5)	724 (11.0)	< 0.001
Day 3 to day 4	10,205	526 (22.1)	955 (12.2)	< 0.001
Day 3 to day 5	8326	450 (24.2)	775 (12.0)	< 0.001
Day 4 to day 5	8326	425 (21.4)	800 (12.6)	< 0.001
Day 1 to any day during ICU stay (any increase)	15,740	1382 (25.7)	954 (9.2)	< 0.001
Day 1 to any day during ICU stay (≥ 2 point increase)^2^	15,740	1124 (32.1)	1212 (9.9)	< 0.001
Day 1 to any day during ICU stay (≥ 3 point increase)^3^	15,740	903 (39.5)	1433 (10.7)	< 0.001

*SOFA* Sequential Organ Failure Assessment

^1^Based on patients who had SOFA scores on both days

^2^Comparison is patients with an increase in SOFA score ≥ 2 vs. patients with an increase < 2 points or a decrease

^3^Comparison is patients with an increase in SOFA score ≥ 3 vs. patients with an increase < 3 points or a decrease

^4^*p* value is based on a chi-squared test

**Fig. 8:**
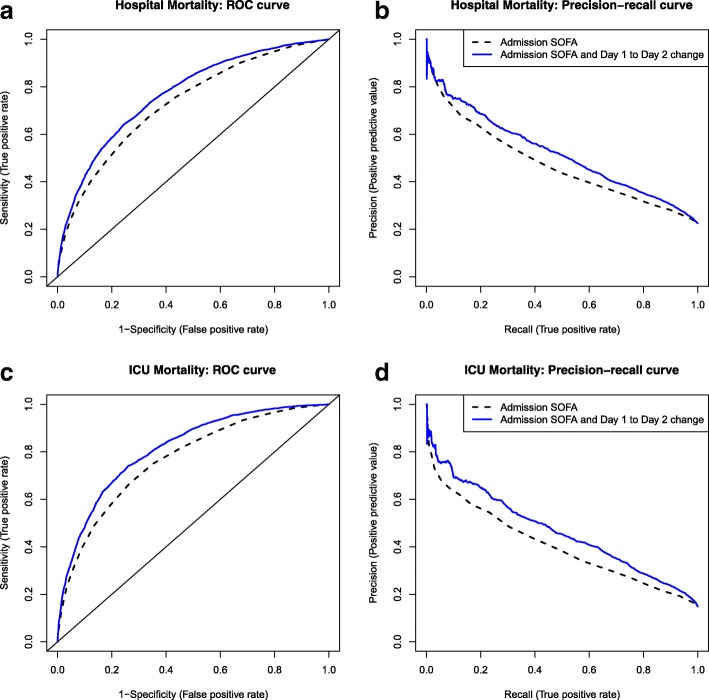
ROC and PR curves for predictors of hospital and ICU mortality. ROC, receiver operating characteristic; PR, precision-recall

Among all 20,007 patients, area under the ROC curve (area under the PR curve in brackets) using admission SOFA as a predictor of hospital mortality was 0.77 (0.57) while it was 0.79 (0.58) using maximum SOFA and 0.81 (0.63) using highest SOFA (Table 5). For ICU mortality, the values were 0.80 (0.53) for admission SOFA, 0.82 (0.54) for maximum SOFA and 0.85 (0.60) for highest SOFA. Area under the ROC curve (area under the PR curve in brackets) was 0.81 (0.62) using the admission APACHE II score for hospital mortality and 0.83 (0.58) for ICU mortality. Among patients with SOFA scores collected on both days 1 (admission) and 2 of their ICU stay (*n* = 15,736), area under the ROC curve was 0.73 using admission SOFA alone, while the difference between days 1 and 2 improved prediction to an area under the ROC curve of 0.77 (*Z* = 13.7, *p* < 0.001). The difference between the admission SOFA and maximum SOFA as well as the difference between the admission SOFA and highest SOFA were also improved with the addition of the admission SOFA (*Z* = 13.6, *p* < 0.001 and *Z* = 17.5, *p* < 0.001, respectively). For ICU mortality, area under the ROC curve was 0.77 using admission SOFA alone while the difference between days 1 and 2 improved the area under the ROC curve to 0.81 (Tables 5 and 6). The ROC and PR curves for hospital and ICU mortality are presented in Fig. 8.

**Table 5:** Area under the ROC and PR curves for predictors of hospital mortality

Predictor	Number of patients	Area under the ROC curve (area under the PR curve)			
		Predictor alone	Admission SOFA score	Predictor + admission SOFA score	Brier score**
Admission SOFA score	20,007	0.77 (0.57)	–	–	0.152
Maximum SOFA score	20,007	0.79 (0.58)	–	–	0.147
Highest SOFA score	20,007	0.81 (0.63)	–	–	0.140
Difference between day 1 and maximum SOFA score	20,007	0.57 (0.37)	0.77 (0.57)	0.79 (0.59)	0.146
Difference between day 1 and highest SOFA score	20,007	0.58 (0.38)	0.77 (0.57)	0.81 (0.63)	0.139
Difference between day 1 and day 2*	15,736	0.57 (0.31)	0.73 (0.47)	0.77 (0.52)	0.143
Difference between day 1 and day 3*	12,650	0.60 (0.33)	0.70 (0.42)	0.76 (0.50)	0.145
Difference between day 1 and day 4	10,206	0.61 (0.36)	0.67 (0.39)	0.75 (0.50)	0.150
Difference between day 1 and day 5	8326	0.62 (0.37)	0.64 (0.38)	0.74 (0.49)	0.157
Difference between day 2 and day 3*	12,648	0.56 (0.29)	0.70 (0.42)	0.71 (0.44)	0.154

*Based on patients who had SOFA scores on both days

**Based on “Predictor alone” for Admission SOFA score, Maximum SOFA score, and Highest SOFA score and based on “Predictor + admission SOFA score” for all other predictors

*ROC* receiver operating characteristic, *PR* precision-recall, *SOFA* Sequential Organ Failure Assessment

**Table 6:** Area under the receiver operating characteristic curves for predictors of ICU mortality

Predictor	Number of patients	Area under the ROC curve (area under the PR curve)			
		Predictor alone	Admission SOFA score	Predictor + admission SOFA score	Brier score**
Admission SOFA score	20,007	0.80 (0.53)	–	–	0.117
Maximum SOFA score	20,007	0.82 (0.54)	–	–	0.115
Highest SOFA score	20,007	0.85 (0.60)	–	–	0.106
Difference between day 1 and maximum SOFA score	20,007	0.55 (0.30)	0.80 (0.53)	0.83 (0.55)	0.113
Difference between day 1 and highest SOFA score	20,007	0.58 (0.32)	0.80 (0.53)	0.85 (0.61)	0.105
Difference between day 1 and day 2*	15,736	0.59 (0.25)	0.77 (0.40)	0.81 (0.47)	0.101
Difference between day 1 and day 3*	12,650	0.64 (0.27)	0.72 (0.32)	0.80 (0.45)	0.100
Difference between day 1 and day 4	10,206	0.66 (0.30)	0.69 (0.28)	0.80 (0.44)	0.102
Difference between day 1 and day 5	8326	0.67 (0.30)	0.66 (0.26)	0.79 (0.42)	0.105
Difference between day 2 and day 3*	12,648	0.59 (0.22)	0.72 (0.32)	0.75 (0.36)	

*Based on patients who had SOFA scores on both days

**Based on “Predictor alone” for Admission SOFA score, Maximum SOFA score, and Highest SOFA score and based on “Predictor + admission SOFA score” for all other predictors

*ROC* receiver operating characteristic, *PR* precision-recall, *SOFA* Sequential Organ Failure Assessment

## Discussion

Our data demonstrate that MODS does have a clinical course that can be described on a population basis though with significant individual variation in trajectory. It also demonstrates that this course is different in hospital non-survivors and survivors, and varies in the first 7 days and the next 7 days based on the severity of admission organ dysfunction. Although using a piecewise linear regression model was a simplification to ease in interpretation, less restrictive models showed similar trends. Among survivors, there is less severe organ dysfunction at admission, and a more rapid improvement in SOFA score compared to non-survivors. The rapidity of daily improvement in SOFA scores over the first 7 days is greater in the cohort with an admission SOFA > 11 compared to < 11. However, from days 8–14, there is no statistically significant difference in improvement in daily SOFA scores in non-survivors, compared to survivors. Most patients had their highest SOFA score in the first 2 days of ICU admission. This may suggest that if “multiple hits” are responsible for MODS, hits are not associated with a clinical course of failure-resolution-failure [5].

Our results are similar to those of Moreno et al. [19]. Their study included 1449 patients from 40 ICUs over 4 continents and demonstrated that maximum SOFA had a higher discriminative ability than the admission SOFA or the Delta SOFA (area under the ROC curve (AUC) of 0.847 vs 0.772 and 0.742, respectively). The relationship of maximum SOFA to hospital mortality in our study was essentially similar to the relationship observed of maximum SOFA to ICU mortality from the Moreno study (AUC 0.82 vs 0.847). One of the recommendations by Moreno et al. was that SOFA scores be compared not only against ICU outcome but with longer outcomes such as 30-day or hospital mortality. Our results complement the earlier findings of Moreno et al. and respond to what they consider to be limitations in their own observations. There was a less of a relationship observed between delta SOFA and mortality in our study and that of Moreno (AUC 0.58 vs 0.742). These differences might reflect differences in the study cohorts, or potential overlap in the calculation of our admission and day 1 SOFA, or that the relationship of delta SOFA to hospital outcome is not as relevant as the relationship with maximum SOFA. It may also suggest that overall trajectory within ICU is a better descriptor of the relationship with hospital mortality. Bingold et al.’s [20] examination of SOFA scores were limited to scores on admission and at 3 days after admission. While Badawi et al. [34] was a much larger study, our patient cohort differed in illness severity with a mean SOFA score of 7.0 in our cohort and a mean SOFA score of 4.5 in their cohort. Additionally, ICU mortality (18.5% vs 4.8%) and hospital mortality (25.5% vs 8.1%) were significantly higher in our patient cohort, and our data was provided beyond 7 days in ICU. These results are also similar to data from Badreldin and colleagues who suggested that a “daily-mean-SOFA” (an average daily SOFA from day 1 to up to day 6—essentially a slope) was more accurate in describing outcome than admission or Max SOFA scores [32]. Holder et al. [33] showed that early serial organ failure scores up to 5 days improve prediction of ICU mortality; however, ICU and hospital mortality rates were very low (6.4% and 11.2%, respectively) in comparison to our study (18.5% and 25.5%, respectively), and coronary care units were included. In spite of including a different study population, the main results from our current study are remarkably similar to those from an earlier study by our group [31]. The current study included 20,007 patients admitted to ICUs in Calgary, Canada, over 10 years (2004–2013), whereas our earlier study included only 1436 patients admitted to Calgary ICUs over 1 year from May 1, 2000, to April 30, 2001. The main difference between the current study and our previous study is the examination in the current study of how SOFA scores change early during ICU admission and the association of these changes with survival. Taken together with our previous study, we have demonstrated that multiple organ dysfunction does not follow a course of progressive and sequential failure. Both studies have also shown that the clinical course of organ dysfunction differs between hospital survivors and non-survivors, but we were additionally able to show this difference varies by admission SOFA and the temporal change in the first week of the patient’s ICU stay. This information is important as it has potential implications for evaluation of therapeutic interventions in the critically ill as has been suggested in recent critical care literature [15, 47–50]. Temporal change in organ dysfunction could be considered as a measure for guiding therapy. For example, in a clinical trial, the absence of improvement in organ dysfunction over a number of days might suggest the absence of benefit, whereas the rate of organ improvement as measured by SOFA might determine the duration of therapy. Decisions to terminate or duration of therapy might be particularly relevant for therapies with high acquisition costs, or for therapies with potentially high risk from adverse side effect profiles.

In addition to demonstrating its potential utility as a tool to guide therapy, our study suggests that a certain “fatalism” about the severity of organ dysfunction may not be justified [18, 19, 51, 52]. Patients in our study with SOFA admission, SOFA highest, or SOFA maximum scores in the 15–19 range and even 20 or above did not have a certain outcome of death (e.g., survival 22.9% for admission SOFA of 15–19 and 5.6% for admission SOFA of 20–24). Our current results expand on our previous observations and conclusions. Careful consideration of other factors, such as admitting diagnosis, frailty, and underlying burden of illness, though not part of our study, could reasonably be evaluated in future work and should be considered in the meantime prior to applying SOFA scores as means of “triaging” outcome.

Our study is not without limitations. First is a potential limitation and criticism of the SOFA score. The original score and cutoff points within each organ system were developed by consensus rather than by mathematical modelling where the interval between ordinal scores purports a proportional increase in the risk of death. Our results do not support such criticism. Whether examining admission, highest, or maximum SOFA, there is an approximately linear increase in the risk of death as SOFA rises. The association of organ dysfunction with outcomes is complex. Although we have reported predicted probabilities of mortality associated with different measures of SOFA (i.e., maximum SOFA), we do not consider that simple mathematical models can fully explain the association, nor are we confident that organ failure scores will provide robust estimates for prognosis. Rather, we believe that this information is descriptive and must be placed in the context of a patient’s individual course. Alternate methods of examining the association have also not provided results that can be used with confidence for predicting outcome [53–58]. A major limitation of this study is the presence of informative censoring of patients who die and are discharged that is dependent on organ function. This was shown in the analysis separating patients into three groups based on ICU length of stay where the profiles differed between the three groups of patients. Further work needs to be done to more closely examine this strong feature of our data. Another limitation arises from our subgroup analyses. Grouping SOFA scores into two categories (≤ 11 versus > 11) is a form of extreme rounding. Although this was done based on previous studies showing increased mortality with SOFA scores > 11 and to show that patients admitted to ICU with a greater degree of organ dysfunction behave differently than those who have low organ dysfunction, caution is advised as simple dichotomization can result in spurious inferences in interpretation.

## Conclusion

Multiple organ dysfunction syndrome is a common problem experienced by many patients admitted to ICUs. The severity of organ dysfunction is usually worst at admission. Death is more common in those with more severe admission SOFA scores. There are clear differences in the rates of improvement in SOFA between survivors and non-survivors with the most rapid improvement in survivors. A lack of improvement in SOFA scores beyond 7 days is the temporal course experienced by most non-survivors of critical illness.

## Additional file

Additional file 1: **Table S1.** Exact *p* values for hospital and ICU mortality by early change in daily SOFA scores. (DOCX 19 kb)

## Abbreviations

CI: Confidence interval
CNS: Central nervous system
GCS: Glasgow Coma Scale
GEE: Generalized estimating equations
ICU: Intensive care unit
IQR: Interquartile range
MODS: Multiple organ dysfunction syndrome
PR: Precision-recall
ROC: Receiver operating characteristic
SOFA: Sequential Organ Failure Assessment
