# Supplementary material for: Describing organ dysfunction in the intensive care unit: a cohort study of 20,000 patients
Source: Crit Care. 2019 May 23;23:186. doi: 10.1186/s13054-019-2459-9 (PMC6533687; doi:10.1186/s13054-019-2459-9)
Supplement: Supplementary file 1 — Table S1. Exact p values for hospital and ICU mortality by early change in daily SOFA scores. (DOCX 19 kb) [file 13054_2019_2459_MOESM1_ESM.docx]

**Supplemental Table 1: Exact p-values for hospital and ICU mortality by early change in daily SOFA scores**

|  | **Exact p-value^4^** | |
| --- | --- | --- |
| **Change in SOFA scores^1^** | **Hospital Mortality** | **ICU Mortality** |
| Day 1 to Day 2 | 3.4 x 10^-54^ | 3.8 x 10^-68^ |
| Day 1 to Day 3 | 7.6 x 10^-65^ | 1.0 x 10^-91^ |
| Day 1 to Day 4 | 6.8 x 10^-60^ | 1.4 x 10^-86^ |
| Day 1 to Day 5 | 9.9 x 10^-58^ | 1.6 x 10^-88^ |
| Day 2 to Day 3 | 2.4 x 10^-30^ | 2.8 x 10^-41^ |
| Day 2 to Day 4 | 2.9 x 10^-45^ | 2.3 x 10^-64^ |
| Day 2 to Day 5 | 9.1 x 10^-54^ | 5.1 x 10^-75^ |
| Day 3 to Day 4 | 4.4 x 10^-33^ | 4.5 x 10^-33^ |
| Day 3 to Day 5 | 4.0 x 10^-36^ | 1.7 x 10^-39^ |
| Day 4 to Day 5 | 9.8 x 10^-20^ | 6.1 x 10^-22^ |
| Day 1 to any day during ICU stay (any increase) | 9.8 x 10^-140^ | 1.1 x 10^-168^ |
| Day 1 to any day during ICU stay (≥ 2 point increase)^2^ | 2.3 x 10^-178^ | 9.7 x 10^-233^ |
| Day 1 to any day during ICU stay (≥ 3 point increase)^3^ | 4.2 x 10^-207^ | 4.6 x 10^-282^ |

Abbreviations: SOFA, sequential organ failure assessment

^1^Based on patients who had SOFA scores on both days

^2^Comparison is patients with an increase in SOFA score ≥ 2 vs. patients with an increase < 2 points or a decrease

^3^Comparison is patients with an increase in SOFA score ≥ 3 vs. patients with an increase < 3 points or a decrease

^4^p-value is based on a chi-squared test
